# Association between immune checkpoint inhibitors and the risk and prognosis of uveitis: a meta-analysis

**DOI:** 10.3389/fimmu.2026.1833351

**Published:** 2026-07-15

**Authors:** Qin Li, Yan Mei, Ya Liu, Xia Li, Yuqin Wang, Chunyan Zhou, Wenlian Mou

**Affiliations:** 1 Department of Ophthalmology, Chongqing Hospital of Traditional Chinese Medicine, Chongqing, China; 2 Department of TCM Comprehensive Treatment Area, Chongqing Hospital of Traditional Chinese Medicine, Chongqing, China; 3 Department of Dermatology and Aesthetic Medicine, Chongqing Hospital of Traditional Chinese Medicine, Chongqing, China; 4 Department of General Surgery, Chongqing Hospital of Traditional Chinese Medicine, Chongqing, China

**Keywords:** immune checkpoint inhibitors, meta-analysis, prognosis, risk, uveitis

## Abstract

**Background:**

Immune checkpoint inhibitors (ICIs) activate antitumor immunity by targeting immune checkpoint molecules such as cytotoxic T-lymphocyte-associated antigen 4 (CTLA-4), programmed death receptor 1 (PD-1), and programmed death-ligand 1 (PD-L1). They have emerged as a key therapeutic modality for multiple malignancies. Nevertheless, excessive immune activation may trigger a spectrum of immune-related adverse events (irAEs). Though uncommon, uveitis is a sight-threatening irAEs that can result in permanent visual loss. The risk and prognostic outcomes of ICIs-associated uveitis remain poorly defined to date. Therefore, we performed this meta-analysis to systematically assess the correlation of ICIs therapy with uveitis risk and prognosis, with the goal of providing evidence-based recommendations for clinical identification and management of this ocular complication.

**Methods:**

We searched PubMed, Embase and Web of Science for cohort studies investigating the association between ICIs therapy and uveitis risk as well as prognosis. The search period was from the database inception to March 2026. The risk of bias of the included studies was assessed with the Risk of Bias in Non-randomized Studies of Interventions (ROBINS-I). The meta-analysis was conducted using RevMan software.

**Results:**

In total, five studies comprising 277,790 participants were finally included. The meta-analysis revealed that ICIs therapy was associated with a significantly higher risk of uveitis (HR = 2.26, 95% CI: 1.46 - 3.51, P = 0.0003). However, no statistically significant association was observed between ICIs-associated uveitis and overall survival in the pooled analysis of two studies (HR = 0.69, 95% CI: 0.35 - 1.39, P = 0.30);this finding is based on limited evidence and should be interpreted with caution.

**Conclusions:**

Current evidence indicates that ICIs therapy was associated with an increased risk of uveitis. However, existing evidence regarding the impact of ICIs-associated uveitis on overall survival is of very low certainty and associated with a high risk of bias, which precludes drawing reliable conclusions. Large-scale prospective multicenter real-world cohort studies are therefore warranted. Such studies should implement standardized ophthalmological assessments and uniform outcome definitions, systematically record anatomical subtypes and severity grades of uveitis, and clarify the relationships between these subtypes/grades and oncologic prognosis as well as systemic immune-related toxicity. Study outcomes should be reported stratified according to cancer type, and further research is required to explore biomarker-based risk prediction.

**Systematic Review Registration:**

https://www.crd.york.ac.uk/PROSPERO/view/CRD420261341882, identifier CRD420261341882.

## Introduction

In recent years, tumor immunotherapy based on immune checkpoint inhibitors (ICIs) has made remarkable advances (1). ICIs target negative immune regulatory molecules including cytotoxic T lymphocyte-associated antigen 4 (CTLA-4), programmed death receptor 1 (PD-1) and programmed death-ligand 1 (PD-L1). These agents reactivate exhausted T cells and enhance the antitumor immune response (2). They have yielded significant survival benefits in multiple advanced malignancies, such as melanoma, non-small cell lung cancer and renal cell carcinoma (3–5). Along with the expanding indications of ICIs and their wider clinical application, immune-related adverse events (irAEs) have become a major concern and clinical challenge in patient management (6).

IrAEs can affect multiple organ systems. Although ocular irAEs are relatively uncommon, they have garnered substantial clinical attention owing to their potential to induce irreversible blindness (7). As one of the most common ocular irAEs, uveitis typically manifests with ocular redness, eye pain, photophobia, and blurred vision. In severe cases, it may result in secondary glaucoma, macular edema, and even irreversible visual loss, severely compromising the quality of life of cancer patients (8–10). Current evidence indicates that the pathogenesis of ICIs-associated uveitis may rely on the cross-recognition of melanin-associated antigens or retinal autoantigens by overactivated T cells (11, 12).

However, existing evidence regarding the risk of uveitis following ICIs therapy remains inconsistent across published studies. Several retrospective cohort studies have demonstrated that the incidence of uveitis is significantly higher in among patients receiving ICIs therapy than in the general population or among cancer patients not receiving such therapy (13). In contrast, other studies have reported a limited absolute increase in uveitis risk, considering its relatively low baseline incidence (11, 14). Furthermore, a more clinically controversial issue is whether the development of ICIs-associated uveitis reflects enhanced antitumor immune reactivity, thereby conferring prolonged overall survival (OS) in cancer patients. Theoretically, the development of irAEs typically indicates robust systemic immune activation and may correlate with superior tumor control (15). However, as a severe local inflammatory complication, uveitis frequently requires systemic or local high-dose glucocorticoid treatment, which may partially attenuate the antitumor efficacy of ICIs (16, 17). Current cohort studies exploring the correlation between uveitis and survival outcomes among cancer patients are limited by small sample sizes and inconsistent findings. Two key clinical questions remain unresolved: whether ICIs therapy elevates uveitis risk and whether ICIs-associated uveitis affects long-term survival. Notably, large-scale data addressing these issues and corresponding systematic meta-analyses are currently lacking. Therefore, the present meta-analysis synthesized available evidence from cohort studies to quantitatively evaluate the association between ICIs therapy and uveitis risk, and to further explore the prognostic impact of ICIs-associated uveitis on OS. This study aims to provide evidence-based guidance for the early identification of high-risk patient populations and for the balanced management of antitumor efficacy and ocular toxicity in clinical practice.

## Methods

This meta-analysis was performed in accordance with the Preferred Reporting Items for Systematic Reviews and Meta-Analyses 2020 (PRISMA 2020) guidelines (18).This study protocol was prospectively registered in PROSPERO (CRD420261341882).

### Data sources and search strategy

A comprehensive literature search was performed across PubMed, Embase, and Web of Science from database inception to March 13, 2026. Manual screening of the reference lists of included studies and relevant systematic reviews was further conducted to retrieve additional eligible literature. The search strategy combined free-text keywords and standardized subject headings, covering “immune checkpoint inhibitors”,”PD-1 inhibitor”,”PD-L1 inhibitor”, “CTLA-4 inhibitor”, “nivolumab”, “pembrolizumab”, “ipilimumab”, “atezolizumab”, “durvalumab”, “avelumab”,”uveitis”,”iritis”,”iridocyclitis”,”chorioretinitis”,”vitritis”,”ocular inflammation”, etc.

### Inclusion and exclusion criteria

Studies were included if they met all of the following eligibility criteria:

1. Study Design: published prospective or retrospective cohort studies with full-text articles available in English;
2. Participant: adult or pediatric patients with pathologically confirmed malignancies;
3. Exposure factor: receipt of ICIs-based monotherapy or combination therapy;
4. Control: patients receiving non-ICIs-based antitumor treatment or no active ICIs treatment;
5. Outcome:

①Primary outcome: the risk of uveitis assessed by hazard ratio (HR) with 95% confidence interval (CI). Uveitis events were defined as newly diagnosed cases during ICIs therapy(excluding patients with a prior history of uveitis); studies exclusively reporting the exacerbation of pre-existing uveitis were excluded.

②Secondary outcome: OS of cancer patients receiving ICIs therapy, compared between patients who developed ICIs-associated uveitis and those who did not. Eligible studies were required to report HRs with corresponding 95% CIs or provide extractable Kaplan–Meier curves and raw data for reliable HR estimation.

Studies were excluded based on the following criteria:

1. Non-peer-reviewed unpublished studies;
2. Studies with insufficient or unextractable data regarding uveitis-related outcomes;
3. Duplicate publications with overlapping study populations;
4. Non-original study types, including case reports, case series, narrative reviews, editorials, and conference abstracts lacking complete raw data.

### Data extraction

Two researchers independently conducted literature screening and data extraction in a blinded manner. Any discrepancies between the two investigators were resolved via consensus discussion, with arbitration by a senior researcher when necessary. Records were first screened by titles and abstracts to exclude obviously irrelevant studies, and the remaining articles underwent full-text evaluation for final eligibility determination. A pre-designed standardized data extraction template was adopted to extract the following information:

1. Basic Information: first author, publication year, study region, and study design (prospective or retrospective cohort);
2. Baseline participant characteristics: total sample size, tumor pathological type, and patient age;
3. ICIs exposure characteristics: categories of administered ICIs (anti-PD-1, anti-PD-L1, and anti-CTLA-4 inhibitors);
4. Outcome indicators: median follow-up duration, adjusted HRs with corresponding 95% CIs, and confounding covariates included in multivariable regression models;
5. Original data required for risk of bias assessment.

### Risk of bias assessment

The methodological quality and risk of bias of all included cohort studies was evaluated using the Risk of Bias in Non-randomized Studies of Interventions (ROBINS-I) tool. This tool is tailored to assess non-randomized interventional studies, including prospective and retrospective cohort studies, and encompasses seven predefined bias domains: confounding bias, participant selection bias, intervention classification bias, bias from deviations from intended interventions, missing data bias, outcome measurement bias, and reported result selection bias. Each domain of all included studies was independently evaluated by two researchers. The overall risk of bias was categorized as low, moderate, or serious: low risk indicated low ratings across all domains; moderate risk indicated at least one moderate domain rating without any serious domains; serious risk indicated the presence of at least one serious domain rating. All evaluative discrepancies were resolved through group discussion and senior researcher arbitration to reach a consensus.

### Statistical analysis

All statistical meta-analyses were conducted using RevMan software. HRs and 95% CIs were adopted as the effect sizes to quantify the association between ICIs therapy and uveitis risk. Given that both uveitis incidence and OS represent time-to-event endpoints, HR was selected as the primary effect measure for quantitative pooling. Studies that directly reported adjusted HRs and corresponding 95% CIs were included for pooled analysis. For studies without available HR estimates, survival data were digitized via Engauge Digitizer software, and HRs were subsequently calculated following the established algorithm proposed by Tierney et al. (19). Prior to pooling, all HR values were log-transformed to normalize the skewed distribution, and corresponding standard errors were computed. Study weights were allocated based on the inverse variance statistical method.

Regarding the prognostic analysis of ICIs-associated uveitis on OS, we strictly screened eligible studies by examining whether original analyses had adequately accounted for immortal time bias. Only studies that explicitly controlled for this time-dependent bias were incorporated into the primary OS pooled analysis. Between-study statistical heterogeneity was evaluated using the Chi² test and I². The fixed-effect model was adopted for analyses with low to moderate heterogeneity (P ≥ 0.10 and I² ≤ 50%), whereas the random-effects model was applied for analyses with substantial heterogeneity (20, 21). Sequential leave-one-out sensitivity analyses were performed by excluding individual studies one at a time to verify the stability and robustness of the pooled results. Publication bias was planned to be assessed via funnel plots and Egger’s regression test when the number of included studies was no fewer than ten. Considering the expected small sample size of eligible studies, the final assessment of publication bias was determined based on the actual number of included datasets.

## Results

### Searching results

**Figure 1:**
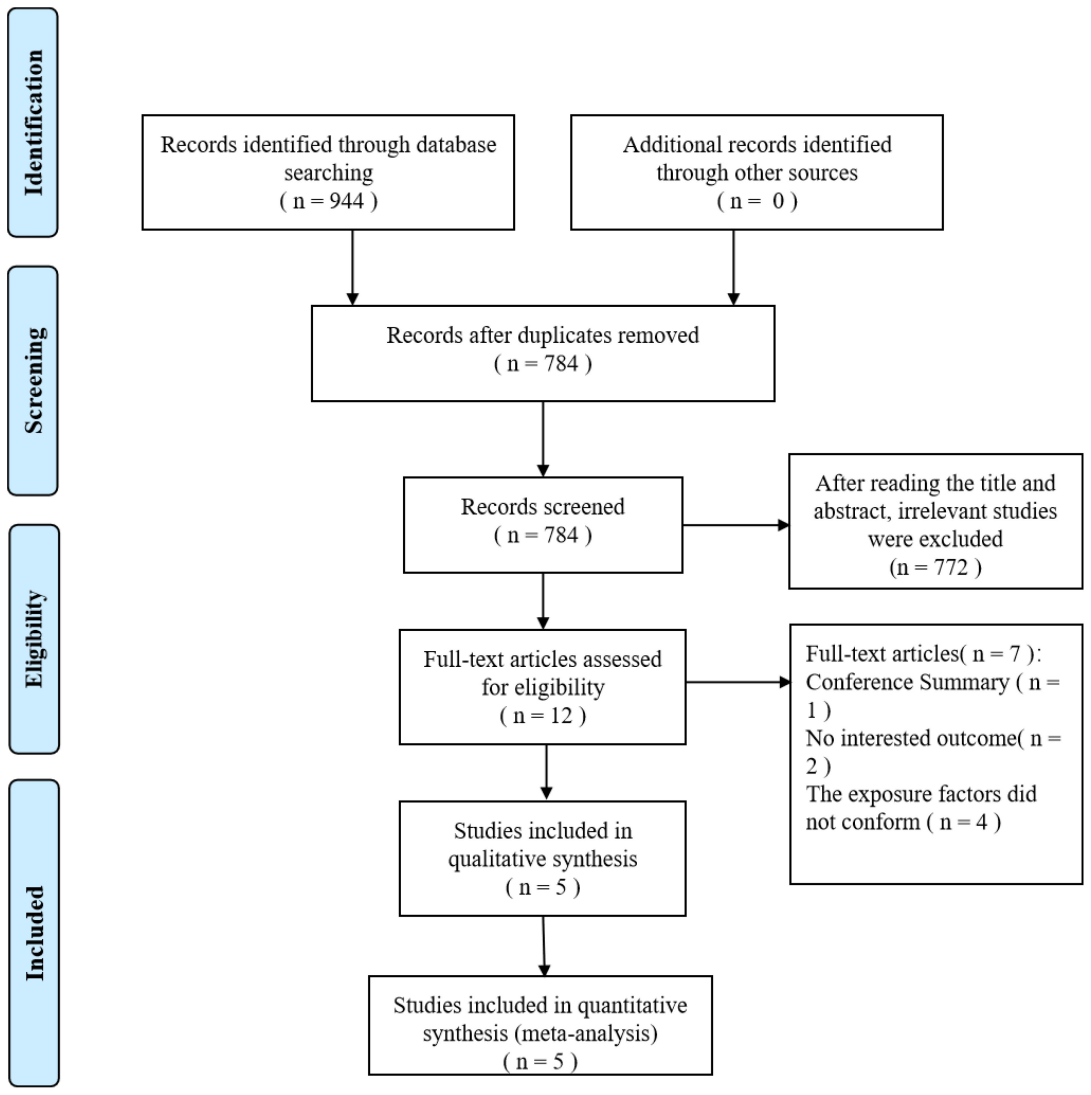
Literature screening flowchart.

The initial database search retrieved 944 articles imported into EndNote. After removing 160 duplicates and multiple rounds of screening, five retrospective cohort studies (11, 13, 14, 22, 23) were finally included, covering a total of 277,790 patients with solid tumors (**Figure 1**).

### Characteristics of eligible studies

The basic characteristics included in the study were shown in **Table 1**. All five studies (11, 13, 14, 22, 23) used a retrospective cohort design, conducted in Asia and North America based on multinational databases, with a total sample size of over 270,000 participants. Published from 2024 to 2025, these studies explored solid malignancies treated with PD-1, PD-L1 and CTLA-4 inhibitors. Most studies adjusted for key confounders with variable adjustment degrees. Follow-up time ranged from 180 days to 5 years. These studies offer high-quality real-world evidence for assessing the risk and prognosis of ICIs-associated uveitis.

**Table 1:** Baseline characteristics of included studies.

Author	Region	Study type	Enrollment period	Tumor type	Case	Age (mean/median)	Types of ICIs	Definition of uveitis	Diagnostic basis	Adjustment factors	Follow-up period
Chang et al. (11)	Korea	Retrospective Cohort Study	From January 2015 to September 2022	Bladder cancer, melanoma	60476	NR	PD-1 inhibitors, PD-L1 inhibitors	New-onset uveitis (Exclude those who have a previous history of infectious or non-infectious uveitis)	ICD-10	Age, sex, Charlson comorbidity index score, and history of autoimmune disease	180,days
Chew et al., 2025 (22)	USA	Retrospective Cohort Study	From January 2015 to March 2024	Cancer	5966	NR	PD-1 inhibitors, PD-L1 inhibitors, TLA-4 inhibitors	New-onset uveitis (Exclude those with a history of non-infectious uveitis.)	ICD-10, ICD-9	NR	Median follow-up was 397 day
Iwai et al. (14)	Japan	Retrospective Cohort Study	From April 2014 to November 2022	Lung cancer, renal cell carcinoma,melanoma	26466	NR	PD-1 inhibitors, PD-L1 inhibitors, TLA-4 inhibitors	New-onset uveitis (Excluding those who already had a diagnosis of uveitis before the start of the treatment)	ICD-10	Age, sex, Cancer type,Brain metastases,Ophthalmological diseases,Comorbidities,Other drug use,Number of eye examinations,and Fiscal year	Median follow-up was 304 days in the ICI group and 425 days in the non-ICI group
Kuo et al., 2024 (23)	Several countries	Retrospective Cohort Study	From January 1–2011 to December 31 2022	Cancer	143862	63.55	PD-1 inhibitors, PD-L1 inhibitors, TLA-4 inhibitors	New-onset uveitis (Exclude those who already had uveitis or related immune/infectious diseases before the treatment.)	ICD-10	Sex, race, comorbidities and medical utilization	5,years
Quiruz et al. (13)	Several countries	Retrospective Cohort Study	From January 1–2011 to December 31 2022	Cancer	41020	65.2	PD-1 inhibitors, PD-L1 inhibitors, TLA-4 inhibitors	New-onset uveitis (Excluding those who had received a relevant diagnosis within 6 months prior to ICI treatment)	ICD-10	Age, sex, race, ethnicity, and cancer type	1,years

PD-1, programmed death-1; PD-L1, programmed death-ligand 1; CTLA-4, cytotoxic T-lymphocyte-associated protein 4; ICI, immune checkpoint inhibitor; NR, Not report.

### Risk assessment of bias

**Figure 2:**
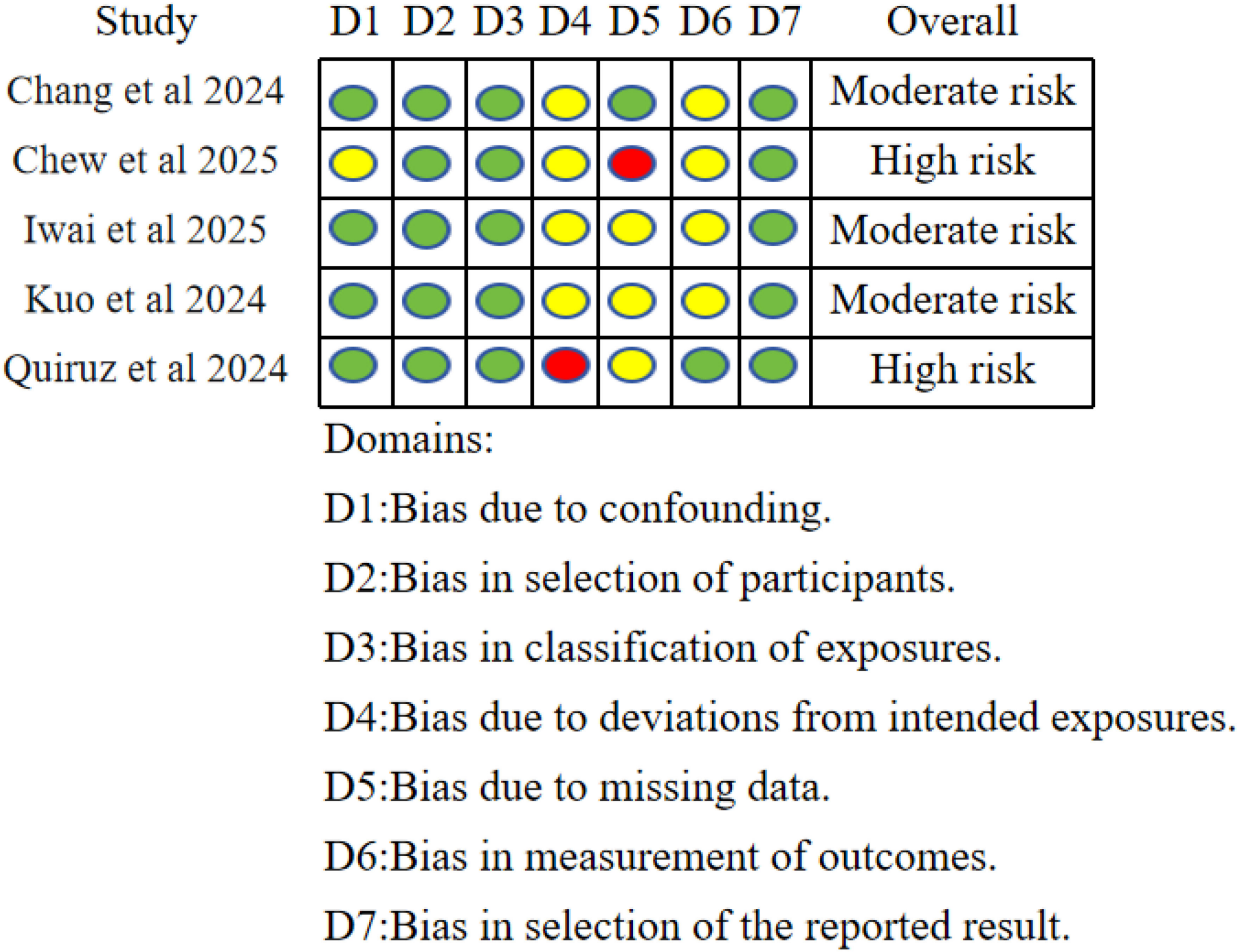
Risk assessment result of bias.

The bias risk of the included studies was evaluated using ROBINS-I (**Figure 2**). Overall, three studies (11, 14, 23) had rigorous methodologies, large sample sizes and adequate control for confounders, with objective outcome definitions, and were rated as having a moderate risk of bias. The remaining two studies (13, 22) were judged to have a high risk of bias due to major concerns across multiple domains, including inadequate confounding adjustment, considerable missing data, single-center design with small sample sizes, outcome ascertainment based on diagnostic codes, and incomplete reporting of survival analysis approaches. These methodological limitations may undermine the reliability of the pooled results.

### Meta-analysis

**Figure 3:**
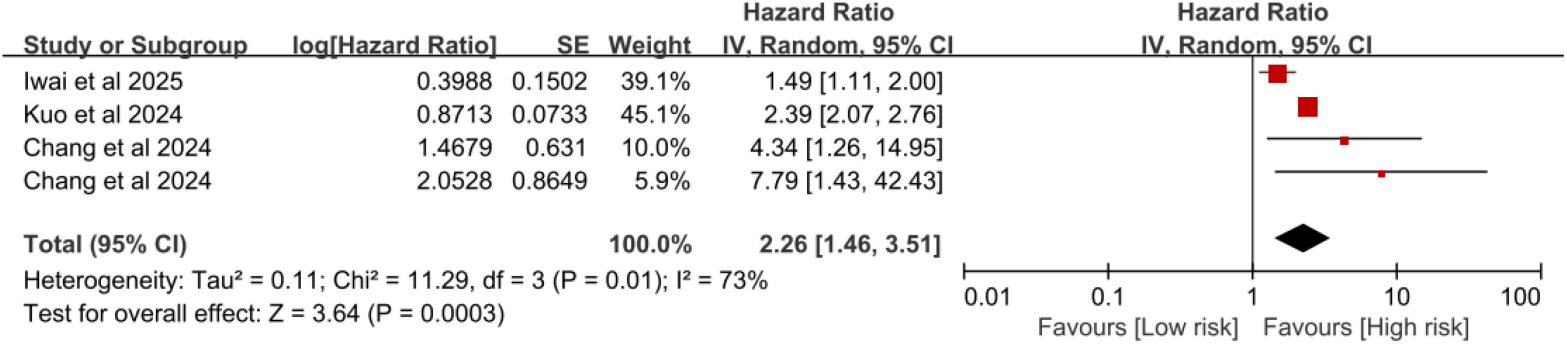
Forest plot showing the association between the use of ICIs and the risk of uveitis.

Four studies explored the association between ICIs therapy and uveitis risk. Substantial between-study heterogeneity was observed (*P = 0.01, I² = 73%*); thus, a random-effects model was applied for meta-analysis. The pooled analysis showed that ICIs therapy was significantly associated with an elevated risk of uveitis among patients with cancer (HR = 2.26, 95% CI: 1.46 - 3.51, *P = 0.0003*) (**Figure 3**).

**Figure 4:**
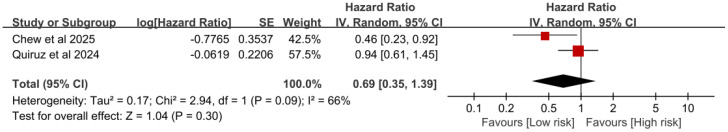
Forest plot showing the association between ICIs-associated uveitis and overall survival.

Two studies investigated the association between tumor patients with ICIs-associated uveitis and those without uveitis. The heterogeneity was detected (*P = 0.09, I² = 66%)*; accordingly, a random-effects model was adopted for meta-analysis. The pooled results revealed no statistically significant association between ICIs-associated uveitis and OS (HR = 0.69, 95% CI: 0.35 - 1.39, P = 0.30) (**Figure 4**). This finding should be interpreted with caution due to the small number of included studies, high risk of bias and existing heterogeneity. The certainty of the evidence is rated as very low, so the result is exploratory and no definitive conclusions can be made.

### Sensitivity analysis

**Figure 5:**
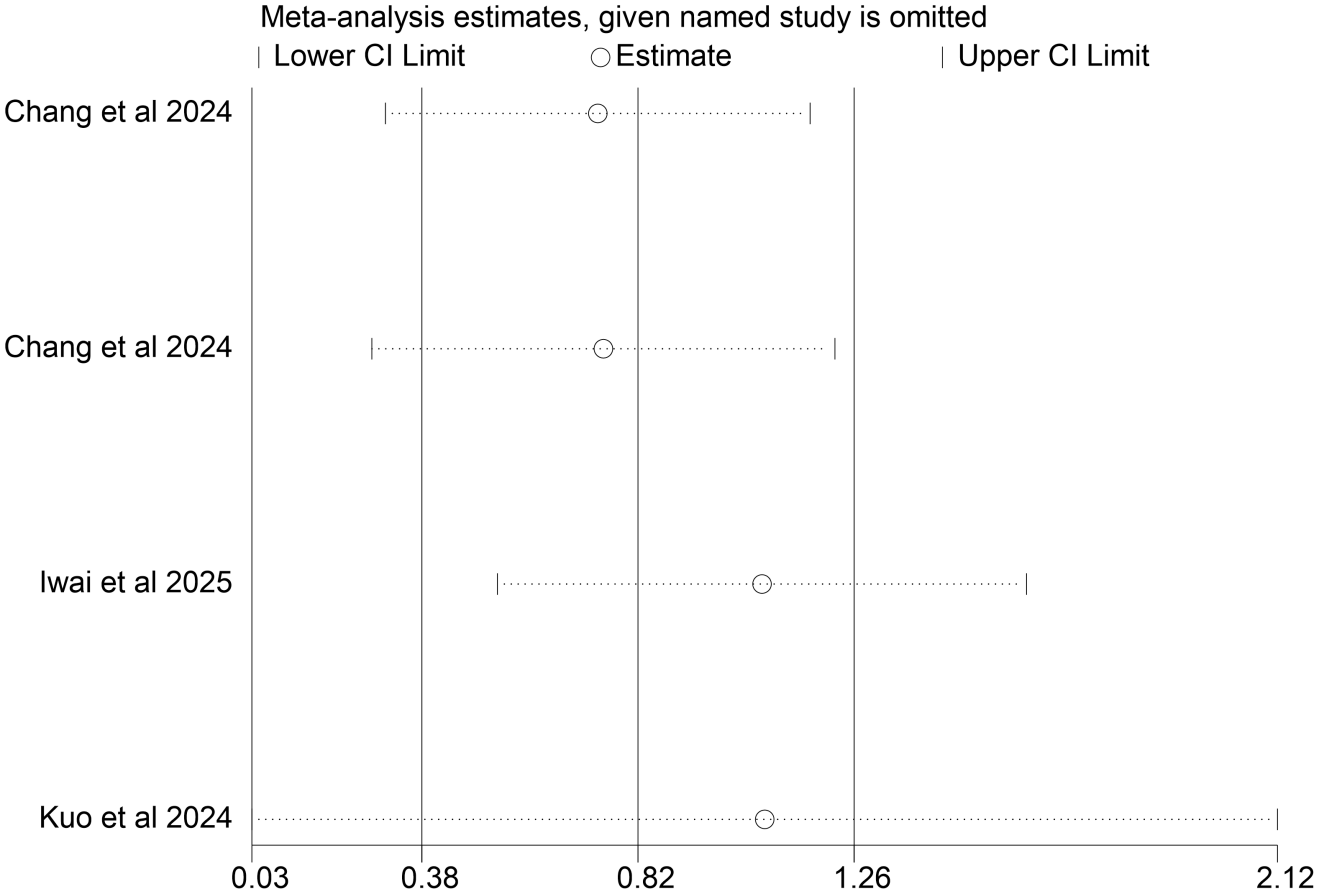
The sensitivity analysis showing the association between the use of ICIs and the risk of uveitis.

A leave-one-out sensitivity analysis was performed to assess the robustness of the association between ICIs therapy and uveitis risk. The pooled estimates remained stable after sequential exclusion of each individual study, confirming the reliability of the meta-analysis results (**Figure 5**).

### Publication bias

Fewer than 10 studies were included in each meta-analysis, so formal tests for publication bias including funnel plot asymmetry assessment and Egger’s test were not conducted. Potential publication bias could not be ruled out, and the conclusions should be interpreted with caution.

## Discussion

To the best of our knowledge, this study is the first systematic evaluation of the association between ICIs therapy and the risk and prognosis of uveitis. The meta-analysis results showed that ICIs therapy was associated with a markedly elevated risk of uveitis in cancer patients, with the risk more than doubled. In contrast, ICIs-associated uveitis had no statistically significant impact on the OS of cancer patients. These findings provide important evidence to guide clinical decision-making for the recognition and management of ICIs-related ocular toxicity.

The pathogenesis of ICIs-associated uveitis shares common features with irAEs, both arising from disrupted self-tolerance driven by excessive T-cell activation following immune checkpoint blockade (10, 12, 24). Mechanistically, irAEs affecting the skin, gastrointestinal tract or eyes reflect a non-specific immune response after the removal of inhibitory checkpoints, and often serve as a pharmacodynamic marker indicating that ICIs have exerted their intended biological effects on the host immune system (25, 26). Consistent with the incidence of irAEs such as rash and thyroid dysfunction, uveitis also demonstrates a dose–response relationship. Notably, patients receiving combined anti-CTLA-4 and anti-PD-1 therapy had a significantly higher uveitis incidence than those on monotherapy (13, 23, 27, 28). Furthermore, most irAEs including uveitis are largely reversible. Early identification and prompt glucocorticoid administration can effectively control inflammation and prevent irreversible organ damage (29–31). This favorable response to immunosuppressive therapy suggests that uveitis shares similar inflammatory pathways with other irAEs, and clinical management strategies are mutually applicable, helping balance the maintenance of antitumor activity and organ protection (12, 30, 32). Accordingly, when managing ICIs-associated uveitis, oncologists may draw on experience with other irAEs to build a rapid referral and multidisciplinary consultation framework, ensuring optimal ocular outcomes and avoiding unnecessary discontinuation of ICIs therapy.

However, ICIs-associated uveitis differs substantially from other irAEs in several key aspects, including organ tropism, predictive value for survival prognosis, and the complexity of therapeutic intervention. First, the eye is an immune-privileged organ characterized by a distinctive immune regulatory microenvironment. The blood–retinal barrier leads to localized inflammatory responses that may not fully reflect the systemic immune status (33, 34). This may partly account for a central finding of the present study: unlike irAEs such as dermatitis and thyroid dysfunction, which are frequently associated with favorable survival outcomes, the development of uveitis did not significantly influence OS in cancer patients. In other words, ocular inflammation may more closely reflect an individual’s susceptibility to eye-specific antigens rather than serving as a reliable surrogate marker for the magnitude of the systemic antitumor immune response. Second, the management of uveitis involves the preservation of vision, a critical function, which frequently necessitates more proactive clinical decision-making. Grade 2 or higher uveitis typically requires topical or systemic glucocorticoid therapy. Prior studies have shown that the dose and duration of glucocorticoid use may potentially attenuate the antitumor activity of ICIs (30, 35). Unlike other organ-specific irAEs, for which alternative strategies can avoid compromising antitumor immunity, excessive suppression of ocular inflammation may inadvertently impair systemic immune function, further weakening the potential association between uveitis and survival benefit. Moreover, the low incidence of uveitis limits the statistical power of large cohort studies to detect modest survival differences, rendering subtle associations difficult to ascertain.

A biological mechanism that warrants particular attention is the antigenic homology shared between melanoma and uveal melanocytes. Following ICIs-induced T-cell activation, autoreactive T cells targeting melanocyte differentiation antigens—such as GP100 and MART-1—may infiltrate the uveal tract, thereby triggering ocular inflammation. Accordingly, ICIs-associated uveitis is anticipated to occur with greater frequency in patients with melanoma and to demonstrate a closer association with antitumor efficacy. Chang et al. (11) reported that the association between ICIs therapy and uveitis reached statistical significance exclusively within the melanoma subgroup, and Chew et al. (22) similarly observed an elevated crude risk of uveitis among patients with melanoma. Notably, this association was attenuated after adjustment for combination immunotherapy regimens. Taken together, these observations suggest that antigenic cross-reactivity between melanoma cells and uveal melanocytes may contribute to the pathogenesis of ICIs-associated uveitis.

Nonetheless, owing to the lack of sufficiently granular subgroup data in primary studies, a formal subgroup meta-analysis restricted to the melanoma subgroup could not be conducted in the present study. In non-melanoma malignancies, which lack melanocytic antigen expression, ICIs-associated uveitis may arise through alternative mechanisms, such as bystander inflammation or cross-reactivity with retinal autoantigens. This tumor-type-specific heterogeneity carries several important implications: (1) the pooled risk estimate derived from this study (HR = 2.26) may not be directly generalizable to non-melanoma populations; (2) the prognostic value of uveitis—specifically, whether it portends a more favorable antitumor response—may vary according to tumor type; and (3) future studies should rigorously stratify analyses by melanoma status and, ideally, incorporate stratification based on specific antigen expression profiles.

These comparative findings carry important clinical implications. First, given that uveitis shares core immune activation mechanisms with other irAEs, clinicians should adopt a holistic, systemic perspective. The development of any irAE in a single organ should raise vigilance for potential inflammatory events across other organs, including the eye, enabling early recognition and coordinated multi-organ management. Second, considering the limited prognostic value of uveitis for survival outcomes, clinical decision-making should not be based on presumed survival benefits when managing ocular inflammation. Instead, treatment strategies should be guided primarily by the principle of organ function preservation. Ocular inflammation should neither be oversimplified as a marker of favorable antitumor response and therefore neglected, nor should ophthalmic intervention be delayed over concerns that glucocorticoids may impair ICIs-mediated antitumor efficacy. A multidisciplinary collaborative framework integrating oncology, ophthalmology, and rheumatology/immunology is highly recommended to facilitate individualized management. For grade 1 (mild) uveitis, ICIs therapy may be continued with close surveillance and tailored topical treatment as needed. For grade 2 or higher (moderate to severe) uveitis, systemic glucocorticoids should be initiated under ophthalmological supervision. Once inflammation is adequately controlled, glucocorticoids should be promptly tapered to optimize the balance between ocular function protection and sustained antitumor efficacy (30, 35). Future studies should focus on identifying specific biomarkers for ICIs-associated uveitis to differentiate ocular inflammation driven by systemic antitumor immune activation from incidental local immune tolerance breakdown. Such biomarkers would support more precise, individualized intervention strategies for this unique immune-related ocular toxicity.

## Limitations

This study has several limitations that should be acknowledged. First, all eligible studies adopted a cohort design. While most had relatively high methodological quality, observational studies are inherently unable to fully eliminate selection and measurement bias, which may affect the robustness of our results. Second, although a random-effects model was applied, variations across studies in ICIs regimens, tumor types, uveitis diagnostic criteria and follow-up duration led to notable clinical heterogeneity. Given the small number of included studies, we were unable to conduct subgroup analyses to identify the sources of heterogeneity. Third, uveitis is a rare adverse event, and the sample size of ICIs-exposed patients in the survival analysis was limited. When assessing the association between uveitis and OS, the wide confidence interval of the pooled HR indicated a potential risk of type II error, meaning a genuine but weak association might have been missed. Although the two primary studies used landmark analysis to reduce immortal time bias, residual confounding remained. Differences in landmark time points (6 months vs. 12 months) and selection bias arising from excluding patients who died before the landmark may have influenced the results. The broad confidence interval and moderate heterogeneity (*I² = 66%*) further suggest that this null finding should be interpreted cautiously. Furthermore, the small number of studies, high risk of bias and existing heterogeneity indicate that the certainty of evidence for this outcome is very low. This result is merely exploratory and cannot support definitive conclusions. The present negative finding should therefore be interpreted with caution, and a significant association may still be detected in larger prospective cohort studies. Fourth, only English-language published articles were included, which may introduce language bias and restrict the generalizability of our findings to non-English-speaking populations. Fifth, none of the primary studies reported uveitis severity graded per Common Terminology Criteria for Adverse Events (CTCAE). Mild and severe uveitis may exert distinct impacts on ICIs efficacy and patient survival. Severe uveitis often requires high-dose systemic glucocorticoids, which can attenuate antitumor immune responses. However, the available data did not allow stratification by disease severity. Finally, no included studies categorized uveitis by anatomical subtype (e.g., anterior, posterior or panuveitis). Different subtypes may arise from distinct immunological mechanisms and present varied systemic toxicity profiles. The lack of such data limits the clinical insights of this analysis and prevents exploration of associations between anatomical subtypes and ICIs efficacy or concomitant systemic irAEs.Sixth,all included studies relied on ICD-10 or ICD-9 codes for uveitis identification, which, although improving case detection consistency, may introduce misclassification bias, particularly when mild or subclinical uveitis is not coded. This may affect the accuracy of the pooled risk estimate.

## Conclusion

In conclusion, this meta-analysis shows that ICIs therapy is significantly associated with an elevated risk of uveitis, highlighting the need for enhanced clinical surveillance. Regarding the prognostic impact of ICIs-associated uveitis on OS, the current evidence is rated as very low certainty and carries a high risk of bias. Thus, no robust conclusions can be drawn so far. Our findings also indicate that intensified monitoring for uveitis is especially necessary among patients receiving combined ICIs therapy. Future research should prioritize large-scale, prospective, multicenter real-world cohort studies with standardized ophthalmological evaluations and unified outcome definitions. Detailed documentation of uveitis anatomical subtypes and severity grades is essential to clarify their associations with oncological outcomes and systemic immune-related toxicities. In addition, results should be reported with stratification by cancer type. Further work is also needed to develop biomarker-based risk prediction models, which can facilitate precision surveillance in clinical practice.

## Data Availability

The datasets presented in this study can be found in online repositories. The names of the repository/repositories and accession number(s) can be found in the article/supplementary material.
